# Maximum predictive power of the microarray-based models for clinical outcomes is limited by correlation between endpoint and gene expression profile

**DOI:** 10.1186/1471-2164-12-S5-S3

**Published:** 2011-12-23

**Authors:** Chen Zhao, Leming Shi, Weida Tong, John D Shaughnessy, André Oberthuer, Lajos Pusztai, Youping Deng, W Fraser Symmans, Tieliu Shi

**Affiliations:** 1 The Center for Bioinformatics and The institute of Biomedical Sciences, School of Life Sciences, East China Normal University, Shanghai, 200241, China; 2 National Center for Toxicological Research, US Food and Drug Administration, 3900 NCTR Road, Jefferson, Arkansas 72079, USA; 3 Laboratory of Myeloma Genetics, Myeloma Institute for Research and Therapy, University of Arkansas for Medical Sciences, Little Rock, AR 72205, USA; 4 MD, Children's Hospital, Department of Pediatric Oncology and Hematology, University of Cologne, Kerpener Strasse 62, D-50924 Cologne, Germany; 5 Department of Breast Medical Oncology and Department of Pathology, The University of Texas M.D. Anderson Cancer Center, Unit 1354, PO Box 301439, Houston, TX 77230-1439, USA; 6 Rush University Cancer Center, Department of Internal Medicine, Rush University Medical Center, Chicago, IL 60612, USA

## Abstract

**Background:**

Microarray data have been used for gene signature selection to predict clinical outcomes. Many studies have attempted to identify factors that affect models' performance with only little success. Fine-tuning of model parameters and optimizing each step of the modeling process often results in over-fitting problems without improving performance.

**Results:**

We propose a quantitative measurement, termed consistency degree, to detect the correlation between disease endpoint and gene expression profile. Different endpoints were shown to have different consistency degrees to gene expression profiles. The validity of this measurement to estimate the consistency was tested with significance at a p-value less than 2.2e-16 for all of the studied endpoints. According to the consistency degree score, overall survival milestone outcome of multiple myeloma was proposed to extend from 730 days to 1561 days, which is more consistent with gene expression profile.

**Conclusion:**

For various clinical endpoints, the maximum predictive powers of different microarray-based models are limited by the correlation between endpoint and gene expression profile of disease samples as indicated by the consistency degree score. In addition, previous defined clinical outcomes can also be reassessed and refined more coherent according to related disease gene expression profile. Our findings point to an entirely new direction for assessing the microarray-based predictive models and provide important information to gene signature based clinical applications.

## Introduction

During the past several decades, scientists have been exploring the relationship between genes and disease [1]. The development of high-throughput gene expression analysis technologies, such as gene-chip and next generation sequencing, has made it possible to understand disease related biological mechanisms at the genome-wide level [2]. From a statistical view, microarray-based clinical applications fall into three distinct types: 1. outcome-related gene discovery, 2. class discovery approaches, and 3. supervised prediction [3]. All these investigations are based on the simple hypothesis that the biological behavior (**= **the individual phenotype) is controlled by the expression level of specific genes [4]. As a severe or even lethal phenotype [5], cancer and many other diseases have drawn enormous scientific attentions, and great efforts have been put forth to identify factors causing the disease on all the levels - from the transcriptome, proteome and regulatory relationship to systems biology [2]. Alternatively, microarray-based approaches have been applied to extract features that can be implemented in a model that accurately predicts various phenotypes of diseases. To this end, many different algorithms, combined with multiple gene signature selection methods, have been applied to generate molecular classifiers [6]. In order to reach higher prediction accuracy, related researchers have been trying to identify different factors that affect each step in model building processes. However, despite fine-tuning approaches and model parameter optimizing strategies, not much significant progress has been made to increase the predictive power of such classifiers. In fact, these explorations revealed considerable over-fitting problems [3].

The MAQC-II Consortium has taken great efforts in attempting to identify factors that affect the accuracy and the consistency of predictive models. Under rigorous control, based on the performance of totally blind external validation to all the endpoints related to human diseases in the three datasets, none of the traditional clinical outcomes can be predicted with outstanding accuracy, except for estrogen-receptor status [7]. In addition, previous published results have also shown that many endpoints in clinical applications cannot be predicted effectively [3].

Many studies have been done with the fundamental assumption that a causal relationship exists between a phenotype and gene expression profile, however, it is very easy to recognize the uncertainty of the hypothesis. First, a common agreement has been reached that the overwhelming majority of intractable complex diseases, especially tumors, are clinically heterogeneous. Moreover, the molecular phenotype may change dynamically both in the process of disease progression and in response to treatment. In microarray-based supervised classification, the predicted endpoints are generally defined by fixed observations at a given time point, such as event-free survival or response to treatment after a given amount of time. As a result, the definition of the criteria to mark off the potentially changing molecular status is entirely based on a factor that may in reality have indefinite values or change in over time, thus affecting prediction performance. With the continuous development of curative techniques, the pathological indexes are progressing synchronously [8,9]. Traditionally, unsupervised hierarchical cluster of a whole expression profile is a descriptive method of the consistency between pathological state and the whole transcriptome state, but is not a quantitative method and far apart from the biological interpretable hypothesis concerning phenotype and gene expression level relationship, which takes only several specific genes into consideration.

**Figure 1:**
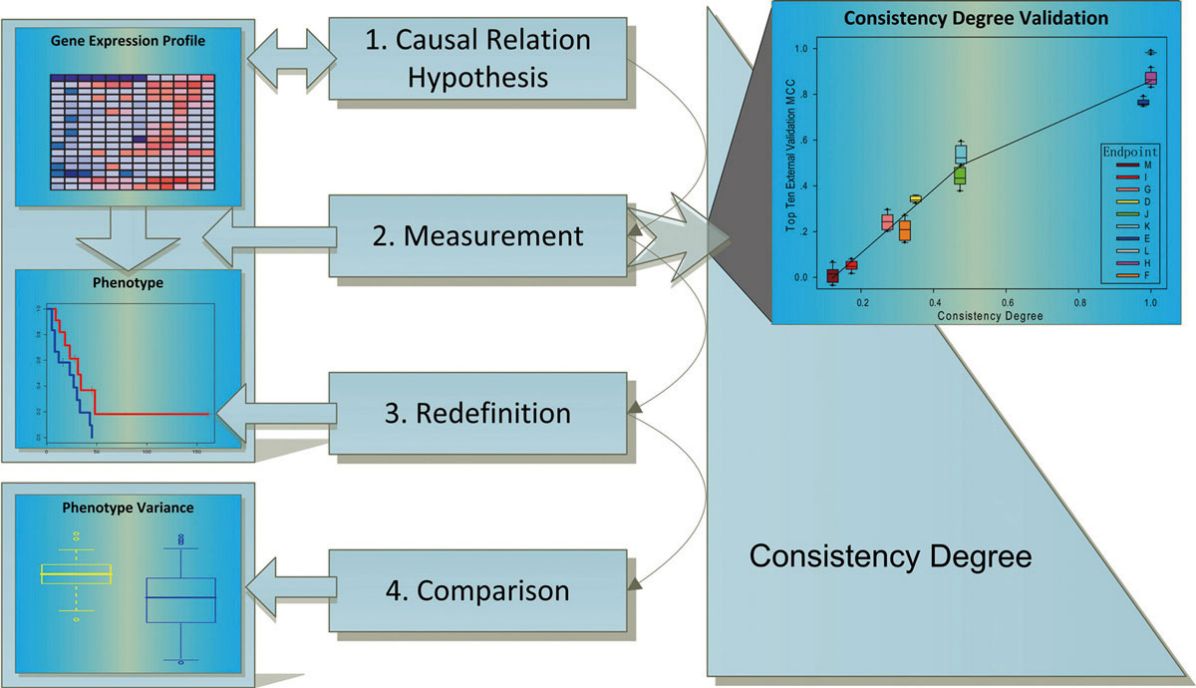
**Overview of analysis flow chart**. 1). All the analysis is based on the hypothesis that a causal relation exists between expression levels of specific genes and an endpoint phenotype. 2) The strength of the relation is calculated by the proposed consistency degree, which is valid with the performance of thousands of models. 3) By maximizing the consistency degree, the endpoint phenotype can be redefined to be most consistent with gene expression levels. 4) Last, traditional and redefined endpoints are compared by the ranges of phenotype variance and the positions of unpredictable samples in gene expression levels.

Here, we propose a quantitative method to describe the consistency between endpoint definition criteria and gene expression profiles and apply it to the three datasets provided by the MAQC-II Consortium. This quantitative degree of consistency, termed consistency degree, is proved to match the classification performance of the external validation in the MAQC-II project [7], and can be used for interpreting, testing and defining the separability of the given endpoint status with regard to gene expression profile (Figure 1).

## Results

### Principle and implementation of the consistency degree

Consistency degree is proposed to be a probability measure to evaluate the degree of the coherent relationship between the endpoint phenotype (pathological criteria) and the gene expression profile. Generally, a microarray-based clinical outcome classification technology is an implementation to fit the relationship between the endpoint phenotype and the gene expression profile. This fitness is entirely based on the hypothesis that a causal relation exists between a phenotype and gene expression profile. Unlike building a classifier, to construct an effective consistency measurement, one not only needs to mine the factors most related to the phenotype, but also needs to take into consideration the corresponding variations within the data. Here, the balance of these two mutual constrained conditions is treated as the principle in the whole procedure. We take advantage of the Spearman's rank correlation coefficient to select potentially phenotype related genes, which have monotonic relation to an endpoint. Since different correlation coefficients have different biases, it is too arbitrary to fix the correlated gene set by just setting a specific p-value in the statistical correlation test. To uncover the largest amount of information concealed within the data in the phenotype related direction, we maximize the variance contribution rates of the first principle component, where the PCA (principle component analysis) or kernel based PCA [10] is recursively performed against a list of genes ranked by descending the calculated correlation coefficients, and the correlated gene set is fixed at the same time. Next, the scores of the first principle component are divided into two groups based on different traits of a defined phenotype. Finally, the consistency degree is defined as the posterior probability of a change point existing between the different traits of a defined phenotype. And the posterior probability is the bootstrapped median of the change point posterior probability calculated by Bayesian change point analysis [11] (additional file 1).

### The validity of the consistency degree

To evaluate the validity of the consistency degree, we calculated it for all of the training datasets about human diseases provided by the MAQC-II project, but not for any of the external validation datasets. We considered the top ten MCCs of all 320 candidate models in the external validation (additional file 2) as a representation of the most reliable and closest relationship between each endpoint and gene expression profile. Then we performed Spearman' rank and Kendall's rank correlation tests between these MCCs and the corresponding consistency degrees (additional file 3). P-values in both of the correlation tests are less than 2.2e-16, proving the validity of this measurement to estimate the consistency between pathological status and gene expression profiles. The consistency degrees for all the change points are shown in additional file 3. Obviously, the consistency degrees of the endpoints I and M are the smallest, the corresponding curves show the weakest changes, and in fact, both of these endpoints are designed to be negative control with the actual class label randomly assigned. In contrast, the consistency degrees of the endpoints H and L, which are designed to be positive control with the actual sex label of the patient assigned [7], are the largest and the corresponding curves have the most significant changes. Besides the endpoints H and L, the endpoint E has the maximum consistency degree (0.979) of the remaining endpoints. As an index of a gene expression product, endpoint E represents the clinical estrogen-receptor status and is easily predictable by microarray-based models. The consistency degrees of the endpoints K and J are in the medium level of the ten endpoints, 0.533 and 0.443, respectively, and endpoint D is relatively lower (additional file 3). The smallest consistency degrees (0.206 and 0.246, additional file 3) are found in endpoints F and G in all of the actual pathological trait defined endpoints. The orders of the consistency degrees for the ten endpoints are significantly related to the average of the top ten MCC of the independent external validation, by which the validity of the consistency degree is distinctly identified. Thus, the consistency degree, which is calculated solely from the training datasets, is definitely valid for the application of interpreting, testing and defining the gene expression profile separability of the given pathological status.

### Redefining the pathological criterion based on gene expression

Besides testing the correlation between each endpoint and gene expression profile, we calculated the consistency degree for any possible cutoff and redefined the pathological criterion for each endpoint to make it as consistent as possible to the gene expression status by iterative reusing the method we proposed. Four endpoints F, G, J and K were redefined here according to the results of our analysis (additional file 4). The endpoints F and G refer to overall survival milestone outcome (OS) and event-free survival milestone outcome (EFS) of multiple myeloma (MM) data set, respectively, whose cutoffs are both set up as 730 (2*365) days. The endpoints J and K, correspond to the outcomes of neuroblastoma (NB), whose cutoffs are both offered as 900 days [7]. According to our algorithm, the resulting cutoffs of G, J and K are close to the given values defined by the MAQC-II project, which are 690, 1,029 and 862 days, respectively, while the cutoff of endpoint F is 1,561 days, which is much larger than the current given cutoff, and all the enrichment p-value of the four cutoffs are less than 2.2e-16 (see methods).

**Figure 2:**
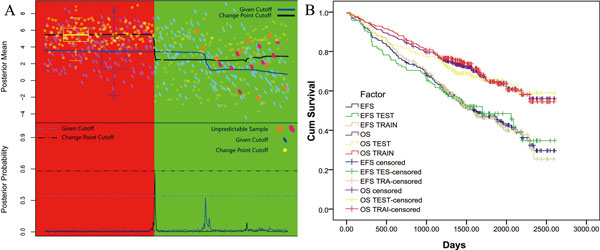
**Change point comparison between the given and redefined cutoffs for endpoint F**. A. **Change point comparison**. Two parts in the figure; the upper one is for posterior mean, and the lower part for the posterior probability. In the upper part, the blue solid lines and the translucent skew ellipses refer to the change point chart of the cutoff 730 days (2*365) given by MAQC-II project, while the others refer to the change point chart with the redefined cutoff 1,561. Posterior mean for the given cutoff is linear scaled to the range of that for the redefined cutoff. The boxplot in the left side of the change point charts represent the ranges of the first component values of the OS negative samples. The larger points represent the unpredictable samples. The skew elliptical ones in purple and the circle ones in orange represent the corresponding positions in the charts with given cutoff and redefined cutoff, respectively. In the posterior probability part of the figure, the dashed lines are the reference lines of the maximum posterior probability values. B. **Kaplan-Meier survival curve**. Six Kaplan-Meier survival curves are shown in the figure for six datasets, including training, test and training-test combined dataset for both EFS and OS endpoint. Three EFS associated curves (blue, green, wheat) are more sloping than OS associated curves (purple, yellow, red) and underneath the OS associated curves.

To evaluate the significance of the new endpoint cutoffs, the bootstrap cutoffs are resampling 1,000 times. Because relationship between cutoffs and gene expression profile is neither simply linear nor continuous, the new cutoffs were not given as in interval estimate, and instead an enrichment test is constructed based on binomial distribution. Furthermore, Kaplan-Meier survival analysis were performed to test the significance of the new endpoint cutoffs, and the result confirms significant difference between OS and EFS outcomes (Figure 2B), the 75 percents survival quantile milestone, purely depending on the clinical data, is quite similar with our non-survival period, which are 729, 1280 days for EFS and OS endpoints respectively (additional file 2). Thus, certain consistence exists between the microarray-based refined endpoints and clinical data, the discrepancy of microarray-based refined endpoints from clinical data can provide potential new reference for reevaluating clinical features on systems level.

Different from the original same given cutoffs, the redefined cutoffs of OS are larger than the cutoffs of EFS in both MM and NB datasets, implying OS days to be a more comprehensive index of samples, which integrate ages, habitus and other factors, while EFS days are more pertinent to the investigated disease. Therefore, the OS is not only larger in the value per se, a larger cutoff of OS can also reflect some other features of a sample [12]. As the result shows, the lesser variance in the overall survival samples indicates that the new cutoffs, which is different from the given criterion, offers us a more coherent gene expression mechanism in the redefined overall survival samples and the larger posterior probability illustrates that the two corresponding traits have a stronger separability based on the gene expression profiles (Figure 2A).

### Significant properties and proposed solution of the unpredictability

To validate our methodology and results, we also used our approaches to analyze those consistently misprediced samples in the MAQC-II independent validation datasets collected from the reported outcome of each team, and found that unpredictability can be explained by the consistency degree score.

**Figure 3:**
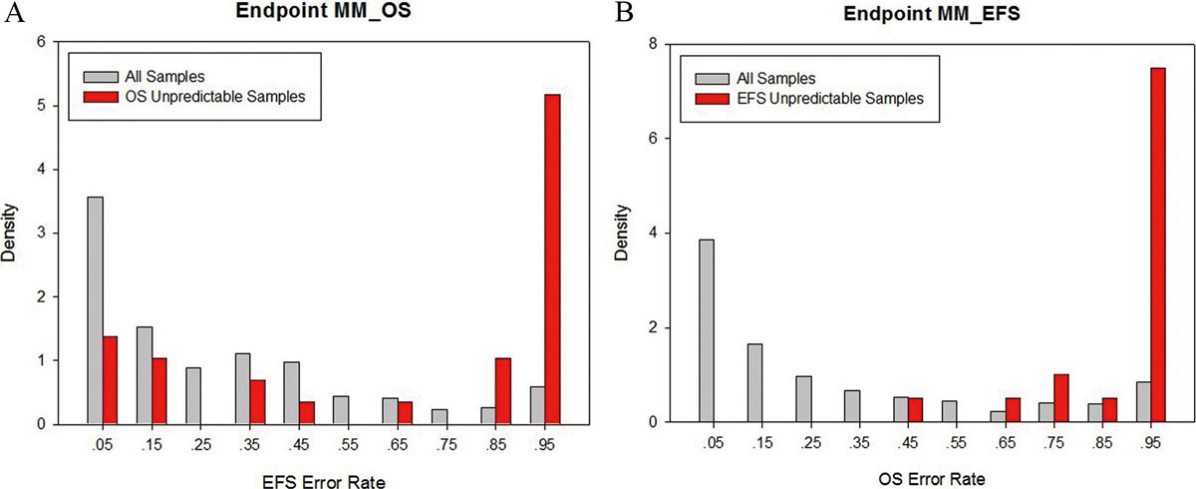
**Unpredictability relationship between endpoint F and G**. A) the cutoff of the error rate to select OS (Endpoint F) unpredictable samples is set to 0.9, and the height of the height of bars represent the probability densities of the samples in corresponding EFS (Endpoint G) prediction error, gray bars for all the samples and red for the OS unpredictable samples with error rate larger than 0.9. Obviously, the OS unpredictable samples tend to have high EFS prediction error rate. B) The same situation presents in the EFS unpredictable samples.

In the consistent misclassification cases of dataset MM, three significant discoveries emerge from our analysis result. First, there is a substantial correlation between the unpredictability of the endpoint F (OS, Overall Survival) and the endpoint G (EFS, Event Free Survival). In other words, the unpredictable samples in the endpoint F are also difficult to be predicted in the endpoint G and *visa versa *(Figure 3). Second, the difference between OS times and EFS times is significantly smaller in the unpredictable samples than that of other samples by the one sided Wilcoxon rank-sum test (p value < 0.05), which indicates that it is difficult to predict those samples that have suffered from severer disease with quick deterioration. Third, for endpoint F, all unpredictable samples (error rate larger than 0.97) were falsely predicted as OS survivors by almost all the predictive models but presented less that 730 days of overall survival. These findings strongly indicate that the clinical standard in defining non-survival in the original endpoint F is too stringent. Although the stringent standard allows for an accurate identification of the non-survival condition (high specificity, top ten average specificity 0.89), meanwhile, it results in misclassifying partial death samples as survival (low sensitivity, top ten average sensitivity 0.31) (additional file 3). Obviously, since the uniformly mispredicted samples are OS positive, the intuitionistic solution for them is to extend the time scale for the non-survival standard and simultaneously narrow the time scale for the survival standard. This is correlated to the result we got based on the correlation from gene expression profile that extends the cutoff of non-survival period from 730 days to 1,561 days.

Next, we adopted the PCA change point method (see methods) to carry out the analysis for those unpredictable samples (see methods) and try to get a deeper insight into the phenomenon of the OS positive uniformly misprediction and the effect of the intuitionistic understandable solution mentioned above. Compared with the given cutoff 730 days, extending the survival time scale to 1,561 days can effectively increase the consistence of survival samples based on the gene expression profile, thereby improving the performance of OS positive (death) prediction (Figure 2A). For the 730 days cutoff given by the MAQC-II project, the first principle component scores of eight out of the nine unpredictable samples are larger than the posterior mean of the scores of the OS positive samples, and fall into the center region of the first component score of the OS negative samples, which is a visible evidence for us to understand the unpredictability (Figure 2A). Relatively, when the cutoff is redefined as 1,561 days, the consistency of the OS negative sample is much more convergent than that of the original given cutoff (the boxplots in the left upper side in Figure 2A). Along with the redefined cutoff, the first principle component scores of six out of the nine unpredictable samples are less than the posterior mean of the score of the OS negative samples, and almost beyond the visibly narrowed dense region of the OS negative samples. This indicates that the redefined cutoff makes it possible to improve the true positive rate for these unpredictable positive samples. From the comparison of the two different cutoffs, it can be derived that the consistency in gene expression profile is not good for the deceased patients' samples (OS = 1). In contrast, the gene expression profiles reach good concordance for the survival samples according to the extended cutoff. At the same time, the re-defined extended cutoff strengthens the correlation between the re-defined endpoint and gene expression profile significantly, thereby providing a solid fundament for building a classifier with higher performance.

## Discussion

The microarray-based technology has drawn more and more attention in the biomedical researches. Numerous experiments have focused on the gene expression profiling generated with microarray technology to better understand the biological mechanisms of disease pathogenesis. Meanwhile, gene signatures selected through microarray data analysis, have been used to predict clinical response, disease stages or subtypes. A lot of investigators have already discussed different aspects of gene signature selection including classification algorithm, producing process, cross-platform comparison, validation, best signature selection.

Feature selection and classifier selection are two core steps in gene expression microarray-based clinical outcome classification for disease. Based on the hypothesis that a causal relation exists between a disease-specific phenotype and corresponding gene expression profile, the feature selection step is considered an exploration of the potential molecular mechanism of endpoints, which is often a time-consuming process. Because of the ambiguity and changeability of disease states under certain criteria, the consistency between phenotype and transcriptome state is instable and may weaken the microarray-based disease related prediction. Furthermore, some pathological standards are defined empirically and restricted to contemporary diagnostic techniques. Since there may be no consistent gene expression profile mechanism underlying a given endpoint phenotype, this indicates that the relationship between a phenotype and gene expression profile should be evaluated prior to exploring a microarray-based predictive model for pathological classification.

Therefore, instead of *a priori *assuming an association between endpoint phenotype and gene expression profile, we propose to first compute the consistency degree to test and evaluate this association. Based on the performances of thousands of classifiers from the MAQC-II project, the validity of the consistency degree was explicitly identified by our study. For example, for the endpoints G, J and K, our results show that the initially attributed cutoff criterion for each endpoint was close to our redefined one, thus indicating a relative consistency between clinical phenotypes and gene expression profiles. However, based on the given criteria, the predictive power of models for those endpoints are still insufficient. This can be reflected by the consistency degree (additional file 3). Since the consistency degrees cannot be increased any more by iterating all of the possible cutoffs, it indicates that there are weak relationships between the pathological traits and gene expression profiles for those three endpoints.

In all of the six actual tested endpoints, endpoint E, refers to estrogen-receptor status, is the only one that can be predicted relatively accurately (average top ten MCC, 0.76, additional file 3). ER is phenotype defined based on the activity of estrogen receptor on the tumor [13] and has clear related molecular mechanism, the consistency degree between this endpoint phenotype and gene expression profile is relative high. The high consistency degree of the endpoint E is a good example to confirm the predictability of the microarray-based models for a pathological endpoint, and is a positive evidence for the existence of the causal relation between gene expression profiles and a pathological endpoint. However, for low predictable endpoints, such as pCR, OS, EFS (additional file 3), which reflect more complicated clinical outcomes resulting from complex clinical treatments, no explicit molecular mechanisms has been elucidated to date. The lower predictive ability of microarray-based model for those complex endpoint phenotypes indicates that the characteristics of those endpoint phenotypes cannot easily be captured by the snapshot of gene expression profile. Therefore, the consistency degree between the phenotype and gene expression profile for those endpoints is much lower. These results imply that there are still wide gaps between complex endpoints and gene expression profile that need to be filled up and the current defined cutoffs for those endpoints need to be further evaluated comprehensively and defined accurately before applying the microarray-based models during clinical applications.

Above all, our results demonstrate that cautions should be taken during the development of microarray-based predictive model and that most importantly, the pathological status need to be carefully examined and defined. Otherwise, enormous effects made by the statistical approach eventually may end up with failure of reaching the ultimate goal, since the maximum predictive power of the models is limited by the correlation between clinical phenotype status and gene expression profile. Based on our findings, we conclude that the consistency degree score is an important index that should be determined before building predictive models based on microarray measurements. Ultimatively, calculating the consistency degree will help to build more reliable classification models.

## Materials and methods

### Datasets

Three of the six datasets and ten of the thirteen endpoints provided by MAQC-II Consortium are used here, including the breast cancer dataset [13], the multiple myeloma dataset t [14], the neuroblastoma datasets [15] and all of the related endpoints of the three datasets [7]. In order to keep the independency between the external validation performance and the consistency degree, none of the external validation data sets are used here.

### Consistency degree of gene expression profile and pathological index

The consistency degree is defined to measure the consistency between endpoint criteria and gene expression profiles. According to the principle, which is the balance of fitting the correlation and keeping the variation, we propose an algorithm which integrate two measurements, the Spearman's rank correlation rho and the contribution rate of the first principle component in PCA, corresponding to the correlation between the gene expression profile and pathological index and the variation consisted in gene expression profile, respectively. An outline of the algorithm is described in additional file 1. According to the algorithm, the consistency degree is represented as the posterior probability of a change point existing between the different traits of a phenotype, calculated by a Bayesian change point analysis [11,16]. Although it is a more simple change point problem in the binary partition situation here, we also perform the fast Bayesian change point analysis [16,17] to obtain a global result. Following Barry and Hartigan [11,16,17], the posterior probability of a change point at position *i*+1 is defined as:

$$p_{i}=P\left(U_{i}=1|X,U_{j},j\neq i\right)$$

and the odds of a change point at a particular position in the partition *i*+1 is:

$$\frac{p_{i}}{1-p_{i}}=\frac{P\left(U_{i}=1|X,U_{j},j\neq i\right)}{P\left(U_{i}=0|X,U_{j},j\neq i\right)}$$

where *U_i _*is an indicator function to indicate where a change point existed in position *i*, and ***X ***is the data. All the associated parameters are the default values [16]. After the odds are maximized, two vectors are estimated, containing the posterior means and the posterior probabilities of a change point at each position. Because the change point problem only contains binary partition situations, and the samples within a partition are randomly ordered, the posterior probability at the position of the boundary between the two types of a phenotype is defined as bootstrapped median of the change point posterior probability calculated by Bayesian change point analysis. The bootstrap resampling was simulated 10,000 times for all the cases here.

### Cutoff selection and endpoint redefinition

Generally, pathological criteria are fixed by statistic analysis of clinical observations, such as TNM staging and so on. Consistent with the motivation of microarray based classification, but more in depth, the most feasible gene expression related cutoff can be found by maximizing the consistency degree. For an orderable pathological index, any value among the range of such an index can be treated as a possible cutoff, and then the corresponding consistency degree can be figured out. Here, the cutoff with the maximum consistency degree is selected as the cutoff to redefine the endpoint and the classes, which the samples belong to.

To estimate the significance of the redefined endpoints, an enrichment test is constructed based on binomial distribution, but not with an interval estimate due to the nonlinear, and discrete relationship between cutoffs and gene expression profile. The significant level *s *is defined as:

$$s\times p=1-F\left(k;n,p\right)=1-\overset{\left|x\right|}{\underset{i=0}{\sum }}\left(\begin{array}{c} n\\ i \end{array}\right)p^{i}{\left(1-p\right)}^{n-i}\;p=1∕N,$$

where *n *(n = 1,000) is the recalculated times of the redefined endpoints, *k *is the recurrence times of a give cutoff, *N *is the number of all the possible cutoffs.

### Unpredictable samples

The list of the prediction error rate for each sample is provided by MAQC-II Consortium. Because none of the external validation data sets are used, all of the samples analyzed here only come from the swap prediction error rate list (additional file 5). In the error rate list, the error rate of the samples, predicted by less than 50 classifiers, are set to be 0.5.

Two error rate cutoffs are fixed to identify the unpredictable samples for the following two different targets. To illustrate the trend of the error rate relationship between the endpoints F and G, the cutoff is set to 0.9 to keep the sample sizes larger than 20. In contrast, when unpredictability is considered, only the best classifiers should be treated as the ones that can reflect the most accuracy relationship between pathological criteria and gene expression profiles, at the point of which, we choose the top ten MCCs to calculate the correlation between the consistency degree and pathological indexes, and only the samples with error predicted numbers less than 20 and with the top ten error rate are selected. The corresponding error rate cutoff is 0.97.

### Statistical analysis

To evaluate the validity of the consistency degree, Spearman' rank and Kendall's rank correlation tests were performed in R [18] with the alternative hypothesis choiced as "greater". One sided Wilcoxon rank-sum test was also performed in the same software to detect the significance of the difference between OS (overall survival milestone outcome) times and EFS (event-free survival milestone outcome) times in the unpredictable samples.

## Competing interests

The authors declare that they have no competing interests.

## Authors' contributions

Data collection: JDS, AO, LP, WFS. Programming: CZ. Design of the analysis process: TLS, LMS, WDT. Data analysis: ZC, TLS. Paper Writing: TLS, CZ, YD, JDS, AO. Paper Finalizing: TLS, CZ, LMS, WDT and YD All authors read and approved the final manuscript.

## Supplementary Material

Additional file 1 **Algorithm of the consistency degree**. Click here for file

Additional file 2 **Performances of the candidate models in MAQC external validation**. Click here for file

Additional file 3 **MCCs and the corresponding consistency degrees**. Click here for file

Additional file 4 **Change Point Redefined Classification Endpoints**. Click here for file

Additional file 5 **Prediction error sample list**. Click here for file
