# Supplementary material for: Maximum predictive power of the microarray-based models for clinical outcomes is limited by correlation between endpoint and gene expression profile
Source: BMC Genomics. 2011 Dec 23;12(Suppl 5):S3. doi: 10.1186/1471-2164-12-S5-S3 (PMC3287499; doi:10.1186/1471-2164-12-S5-S3)
Supplement: Additional file 1 — Algorithm of the consistency degree. [file 1471-2164-12-S5-S3-S1.doc]

| Given a log2 gene expression matrix with m rows and n columns, say matrix *X*. |
| --- |
| Given the phenotype class label for each of columns of matrix *X*. |
| for *i*=1 to *m*, do |
| Compute the Spearman's rank correlation rho, and two-sided test p-values, say *p_i_* |
| End for |
| Compute the ranked row names by increasing the vector *p*, say R. |
| Compute the number of the probes with the corresponding p-value less than 0.01, say K. |
| for i=5 to K, do |
| Pick the sub matrix of X by row names included in the Vector *R*_1_: *_k_* , say *X_i_* |
| PCA or Kernel-based PCA of the picked *X_i_* |
| Compute the variance contribution rates of the first principle component, say *R_i_* . |
| End for |
| Pick the sub matrix with the maximum *R_i_* , say *X_r_* |
| Compute the first component value for each column, say *V* |
| Compute bootstrapped median posterior probability between classes by Bayesian change point analysis |
|  |
